# Robotic Ankle Training Improves Sensorimotor Functions in Children with Cerebral Palsy—A Pilot Study

**DOI:** 10.3390/jcm12041475

**Published:** 2023-02-12

**Authors:** Yunju Lee, Deborah Gaebler-Spira, Li-Qun Zhang

**Affiliations:** 1 School of Engineering, Grand Valley State University, Grand Rapids, MI 49401, USA; 2 Department of Physical Therapy and Athletic Training, Grand Valley State University, Grand Rapids, MI 49401, USA; 3 Shirley Ryan AbilityLab, Chicago, IL 60611, USA; 4 Feinberg School of Medicine, Northwestern University, Chicago, IL 60611, USA; 5 Department of Physical Therapy & Rehabilitation Science, University of Maryland, Baltimore, MD 21201, USA; 6 Department of Orthopaedics, University of Maryland, Baltimore, MD 21201, USA; 7 Department of Bioengineering, University of Maryland, College Park, MD 20742, USA

**Keywords:** robotic training, rehabilitation, sensorimotor function, lower extremity, cerebral palsy

## Abstract

Children with cerebral palsy (CP) have sensorimotor impairments including weakness, spasticity, reduced motor control and sensory deficits. Proprioceptive dysfunction compounds the decreased motor control and mobility. The aims of this paper were to (1) examine proprioceptive deficit of lower extremities of children with CP; (2) study improvement in proprioception and clinical impairments through robotic ankle training (RAT). Eight children with CP participated in a 6-week RAT with pre and post ankle proprioception, clinical, biomechanical assessment compared to the assessment of eight typically developing children (TDC). The children with CP participated in passive stretching (20 min/session) and active movement training (20 to 30 min/session) using an ankle rehabilitation robot (3 sessions/week over 6 weeks, total of 18 sessions). Proprioceptive acuity measured as the plantar and dorsi-flexion motion at which the children recognized the movement was 3.60 ± 2.28° in dorsiflexion and −3.72 ± 2.38° in plantar flexion for the CP group, inferior to that of the TDC group’s 0.94 ± 0.43° in dorsiflexion (*p* = 0.027) and −0.86 ± 0.48° in plantar flexion (*p* = 0.012). After training, ankle motor and sensory functions were improved in children with CP, with the dorsiflexion strength increased from 3.61 ± 3.75 Nm to 7.48 ± 2.75 Nm (*p* = 0.018) and plantar flexion strength increased from −11.89 ± 7.04 Nm to −17.61 ± 6.81 Nm after training (*p* = 0.043). The dorsiflexion AROM increased from 5.58 ± 13.18° to 15.97 ± 11.21° (*p* = 0.028). The proprioceptive acuity showed a trend of decline to 3.08 ± 2.07° in dorsiflexion and to −2.59 ± 1.94° in plantar flexion (*p* > 0.05). The RAT is a promising intervention for children with CP to improve sensorimotor functions of the lower extremities. It provided an interactive and motivating training to engage children with CP in rehabilitation to improve clinical and sensorimotor performance.

## 1. Introduction

One of the most common motor disabilities of childhood is cerebral palsy (CP) [1,2]. Though the primary pathological injury is non-progressive, occurring to a developing immature brain, the cascade of sensorimotor difficulties caused by impairments, such as muscle weakness and spasticity, loss of selective motor control and sensory deficits, create a progressive pattern of neuromuscular dysfunction and biomechanical malalignment [3,4,5,6]. In particular, sensory impairment including diminished proprioceptive acuity affects postural control and development, gait functions and motor activities adversely [7].

Strength is one of the main factors associated with gross motor function in children with CP. Muscle weakness is a key negative symptom of the upper motor syndrome [8]. Weakness in the lower extremities causes the balance and gait impairments of childhood, and furthermore significantly restricts the activities of daily living and impairs quality of life [8,9]. There are studies to evaluate muscle weakness of lower extremities in CP, such as on the knee extensors and flexors, which is typically assessed by manual muscle testing and isokinetic testing. One study showed that children with CP had knee strength deficits, especially knee flexor, and knee flexor strength at different angular velocities was associated with gross motor impairment [10]. Other studies investigated improving muscle strength through conventional exercises (power-training and treadmill training), or physical therapy [11,12,13]. Innovative technological use of devices such as the elliptical machine or a robotic device for patients with muscle impairments have served as new rehabilitation tools for measuring and training human motor skills [14,15,16].

There is evolving evidence that strength is just one aspect of a complex interplay of impairments that affect function. Spasticity, poor alignment, due to a reduced range of motion, loss of selective motor control and reduced sensory perception all impact the ankle in children with CP. In addition, improving the range of motion (ROM) of the ankle has been shown to help improve balance and gait. There have been recent concerns over detrimental effects of neurolytics on the muscle, as potentially contributing to the weakness of the muscles [5]. Utilizing non-pharmacologic physical modalities to improve ROM may offer an advantage for strength training in CP [17].

In many cases, sensory impairments are often accompanied by a disturbance of motor disorders such as strength deficits and limited range of motion. Motor and sensory impairments in children with CP were extensively discussed in the literature, but most of them studied upper limb (hands) functions, and less so lower limb functions [18,19,20]. Recent attention has focused on sensory deficits in light touch, two point discrimination and vibration in the foot and ankle in children with CP and the relationship to balance [21]. The determination of which sensory deficits impact balance and motor function most remain open to exploration. Reduced proprioception has been studied in the forearm [22] and on the hip in children with CP [23] and was found to be an underlying factor for impaired motor control. Goble’s study reported a decreased motor control of the upper extremities due to deficient proprioceptive function among children with CP [22]. Altered proprioception has also been found to occur at the hip in children with CP and may account for gait deviations. However, the sensory deficit of the ankle and foot in children with CP has not been well studied. Proprioception is the sense of self movement and body position, and it is mediated by proprioceptors or mechanosensory neurons located in muscle tendons and joints.

The innovative technological use of devices such as the elliptical machine or a robotic device for patients with muscle impairments have served as new rehabilitation tools for measuring and training human motor skills [14,15,16]. Previous studies utilizing the robotic ankle training (RAT) demonstrated encouraging results to improve ROM, strength, and motor function [14,15]. With the robotic ankle, repeated movement of the ROM is visualized and emphasized as to increase awareness of movement and hence increase proprioceptive practice. Antidotally, following training with the robotic ankle trainer, proprioceptive feedback improved. Therefore, the purpose and rationale of this study was to investigate quantitatively the proprioceptive deficit at the ankle of children with CP, and to explore improvement in sensorimotor functions including proprioception following the robot-guided ankle training.

## 2. Materials and Methods

### 2.1. Participants

Eight children with CP and ankle impairment participated in the study for the robot-guided passive and active movement exercise program. Descriptive characteristics of the participants who completed the exercise program were given in Table 1.

The study inclusion criteria: (i) have a diagnosis of cerebral palsy with diplegia or hemiplegia; (ii) spasticity at the ankle joint with a Modified Ashworth Scale (MAS) > 0; and (iii) the ankle has limited joint mobility. Exclusion criteria included (i) subjects who have other unrelated neurological impairments or musculoskeletal injuries, (ii) patients who have had orthopedic surgery within 6 months prior to participation in the study, and (iii) patients who have severe pain in the impaired limb. The children with CP (either hemiplegia or diplegia) with marked hypertonia were recruited for the study. For the hemiplegic patients, the impaired side was treated and evaluated. For the diplegic patients, the side with more severe hypertonia was investigated. Patients were selected from the patient pool of the Shirley Ryan AbilityLab (formal Rehabilitation Institute of Chicago, RIC) and Northwestern University (NU) physicians.

Eight typically developing children (TDC, age: 11.4 ± 2.4 years, 1 girl and 7 boys) were recruited for a one-time evaluation of ankle proprioception. All eight children with CP completed the total exercise training sessions of combined passive and active movement intervention using a RAT. All participants were able to interact verbally with plain English and they did not have intellectual disabilities. Their parents were also present during the consent procedure. The study was approved by the Institutional Review Board of Northwestern University and all children’s legal guardians gave written informed consents.

### 2.2. Experimental Training Procedures

**Figure 1:**
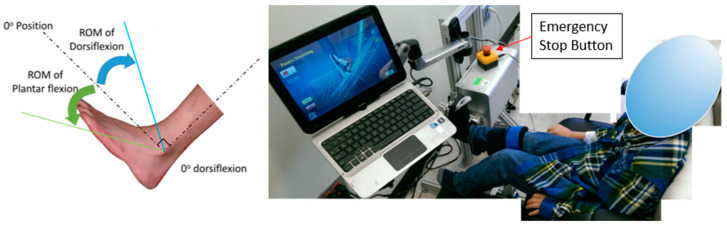
Range of motion (ROM) of ankle movement and definition of 0° dorsiflexion (**left**). Experimental setup (**right**) shows a child in a seated position with an extended knee and an impaired ankle strapped onto a footplate. An ankle rehabilitation robot with operated passive stretching and active movement exercise program and a computer game interface was used for training and outcome measures.

**Figure 2:**
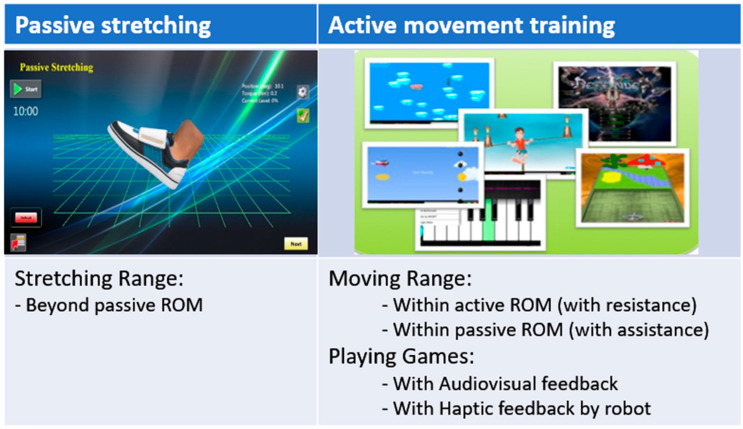
Schematic representation of the training session. Participant of children with CP performed passive stretching (20 min/session) and active movement exercise (20 to 30 min/session) therapy using an ankle rehabilitation robot (3 sessions/week over 6 weeks, total of 18 sessions).

An ankle rehabilitation robot (IntelliStretch^TM^, Rehabtek LLC, Linthicum Heights, MD, USA) with controlled passive stretching and active movement training and a computer game interface was used for the intervention and outcome evaluations [15,24,25,26]. Children with CP received combined robotic training 40 to 50 min per session, 3 sessions per week for a total of 18 sessions on her/his impaired ankle at a research laboratory in the Rehabilitation Institute of Chicago. The children sat securely in a chair with their knee at full extension and foot strapped to a footplate (0° ankle angle as shown in Figure 1). Each training session started with 10 min of passive stretching, followed by performing 20 to 30 min of active movement training, and concluded with 10 min of passive stretching. Figure 2 shows the passive stretching and active movement training procedure of each training session for children with CP. During the active movement training, motivating games with audiovisual feedback was used to engage children with CP. and they played movement games with the robot giving assistance beyond their active ROM and providing resistance within their active ROM to keep challenging the children. 

### 2.3. Outcome Measures and Assessments

Biomechanical and clinical assessments were performed before and after the 18 sessions of training (pre- and post-training). Biomechanical assessments contained the active range of motion (AROM), passive range of motion (PROM), and muscle strength (maximal voluntary contraction (MVC)). AROM in dorsiflexion was quantified by asking patients to voluntarily move the ankle with the foot and footplate from the ankle neutral resting position to maximum dorsiflexion, while obtaining visual feedback of their movement on the computer screen (HP TouchSmart TM2 Notebook PC, Hewlett-Packard, Palo Alto, CA, USA). The average values were calculated to be the AROM in dorsiflexion after three trials were achieved. When the child was asked to dorsiflex maximally, the strength of the dorsiflexor (mainly tibialis anterior) muscles was quantified by locking the footplate at 0° dorsiflexion three times.

Muscle strength of the plantar flexor (gastrocnemius and soleus) muscles was measured three times in the same way, with maximal plantar flexor effort with the footplate locked at 0° dorsiflexion. Each of the three trials of both MVC and AROM assessments were performed at the beginning and the end of each training session. Proprioceptive acuity was quantified as the threshold angle in plantar or dorsiflexion movement (at the speed of 0.5°/s managed by the ankle rehabilitation robot) at which the children noticed the motion.

Demographics (e.g., age, gender, weight) and anthropometrics, disability type and characteristics of the children with CP were recorded and shown in Table 1. Each child completed the following functional evaluations during clinical assessment sessions. The Pediatric Balance Scale (PBS) [27] was used to estimate the balance function. The Modified Ashworth Scale (MAS) [28] was used to measure the calf muscle hypertonia of the participants. The Selective Control Assessment of the Lower Extremity (SCALE) [29] was used to assess selective voluntary motor control (SVMC), which may be useful for assessments of participants with CP. The 10 m walk test (10 MWT) and 6 min walk test (6 MWT) were used to determine the walking capacity of the participants and Timed-Up-and-Go (TUG) was applied for measuring muscle function and the risk of fall as well [30].

### 2.4. Statistical Analysis

Since the assumptions of normality were not always fulfilled, and due to the small sample size, we performed the nonparametric test. We ran Wilcoxon rank sum test to explore the proprioceptive acuity between the children with CP and the TDC. In addition, Wilcoxon signed rank test was applied to compare the baseline measurements (pre-training) with the post training measurements within the children with CP. Biomechanical outcomes obtained by the ankle rehabilitation robot were calculated for each subject and for each training session in multiple trials. All analysis was accomplished using SAS JMP Pro 14 (SAS, Cary, NC, USA). A *p* value of less than 0.05 was considered statistically significant.

## 3. Results

### 3.1. Proprioceptive Deficit

**Figure 3:**
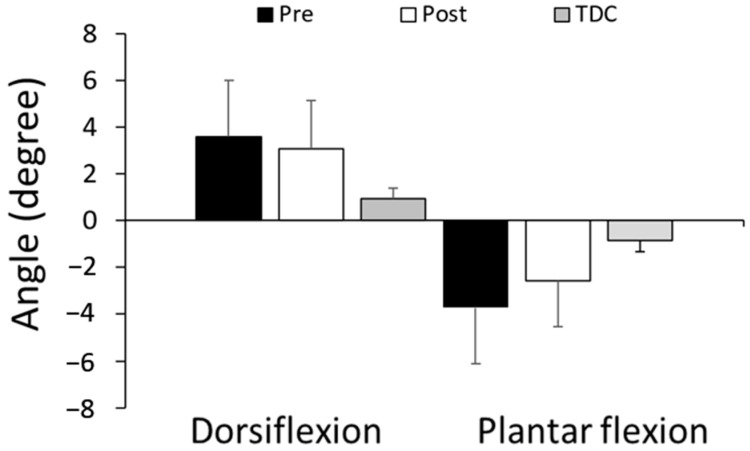
Proprioceptive acuity in dorsiflexion and plantar flexion. Proprioceptive acuity was measured as the threshold angle in plantar or dorsiflexion movement (at the speed of 0.5°/s operated by the ankle rehabilitation robot) at which the participant recognized the movement.

Figure 3 shows that children with CP had significantly impaired proprioception, compared to the typically developing children, at the ankle. The proprioceptive acuity of the CP group was 3.60 ± 2.28° (mean ± SD) in dorsiflexion and −3.72 ± 2.38° in plantar flexion, as compared to that of the TDC group’s 0.94 ± 0.43° (*p* = 0.027) and −0.86 ± 0.48° (*p* = 0.012). Additionally, children with CP sometimes discovered the motion changes in the wrong direction (Table 2).

### 3.2. Effect of RAT

**Figure 4:**
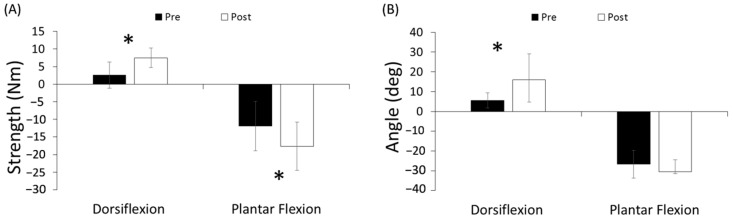
Biomechanical outcome amounts at pre- and post-training. (**A**) shows the strength of dorsiflexor and plantar flexor and (**B**) gives the active range of motion in dorsiflexion and plantar flexion of trained ankles among children with CP. (*) indicates significant difference (*p* < 0.05).

The biomechanical outcome measurements are described in Figure 4. After 18 sessions of the robot-guided passive-active training, ankle sensory and motor functions were significantly enhanced. Trained ankle muscle strength significantly increased both in dorsiflexion (from 3.61 ± 3.75 Nm at pre-training to 7.48 ± 2.75 Nm at post-training (*p* = 0.018)) and in plantar flexion (from −11.89 ± 7.04 Nm at pre-training to −17.61 ± 6.81 Nm at post-training (*p* = 0.043)). A negative torque measurement of dorsiflexor strength means it is lower than the passive torque at 0° dorsiflexion. The AROM in dorsiflexion significantly improved from 5.58 ± 13.18° before training to 15.97 ± 11.21° after training (*p* = 0.028), while AROM in plantar flexion changed from −26.58 ± 5.92° to −30 ± 1.02° over the 6-week exercise training. A negative angle value in dorsiflexion means not being capable of reaching 0° dorsiflexion.

**Figure 5:**
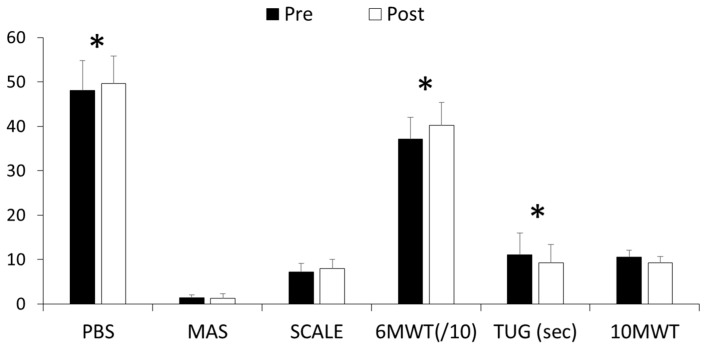
Clinical outcomes before and after training in children with CP. (*) indicates significant difference (*p* < 0.05).

Figure 5 presents the result of assessments in the selective motor control, balance, and walking performance on clinical evaluations at pre-training and post-training. Most of the clinical evaluations showed enhancements between pre-treatment and post-treatment after the 6 weeks of robotic training. Balance function and walking performance in participants with CP was significantly improved in PBS (*p* = 0.026), TUG (*p* = 0.018), and 6 MWT (*p* = 0.018) in Table 3. The score of selective voluntary motor control of lower extremity (SCALE) was increased after RAT, shown as SCALE (*p* = 0.084) in Table 3. The ankle hypertonia for participants with CP was diminished after the 6-week training, as shown by decreased MAS scores; however, these differences were not statistically significant.

Furthermore, the proprioceptive acuity threshold showed enhancement from 3.60 ± 2.38° to 3.08 ± 2.07° in dorsiflexion (*p* > 0.05) and from −3.72 ± 2.38° to −2.59 ± 1.94° in plantar flexion (*p* > 0.05). After RAT, the children with CP detected the movement wrong direction with less errors (Table 2).

## 4. Discussion

We investigated proprioception as a sensorimotor function in children with CP using the ankle rehabilitation robot as a pilot study. First, the results showed that the sensorimotor function, proprioception at ankle, in children with CP was significantly impaired compared to the TDC. Secondly, the study demonstrated the feasible improvement in sensorimotor functions including proprioception, the range of motion, and muscle strength through RAT in biomechanical and clinical measurements.

Convincing data supported the direct positive relationship between motor function and sensation in the upper extremity in children with hemiplegia [31]. Training of proception of the upper limbs has revealed that attention to this impairment may be a strategy to increase upper extremity function [32]. Sensory systems are less frequently sampled in lower extremity research and within the clinical records for children with CP. However, growing evidence supports the inclusion of standardized sensory measures, as well as discriminating which systems impact balance and mobility. In a pilot study, ten children with CP were evaluated with the Balance Evaluation Systems Test (BESTest) and a systematic sensory evaluation [33]. They found that the ability of sensing ankle joint position was significantly associated with the postural responses subdomain of the BESTest. Hence, it is important to note that we focused on the ankle joint in lower limbs, and the current study highlighted that there was a sensorimotor deficit among the children with CP compared to TDC.

Sensing joint position and kinesthetic sensation have been tested in both the arm and leg in children with CP, and it was found that proprioceptive error occurred in the lower extremity, biased to internal rotation [23]. This is consistent with the current observation that the children with CP had more errors than TDC in the proprioception test. Balance and gait are complicated tasks, implying the integration of appropriate sensory and motor systems. It is known that children with CP rely on the visual systems to a greater degree than typical children, and furthermore, children with diplegia used the same muscle groups as typical children in gait, but they used a different strategy, relying more on proximal muscles than TDC [34,35]. Therefore, the improvement in proprioception and muscle strength in lower limbs could help their balance and gait functions. The current results supported this positive relationship, shown as increased strength and range of motion of ankle muscles in biomechanical outcomes (Figure 4), as well as better scores in clinical measurements ( Table 2; Table 3) after RAT.

Since both sensory and motor deficits contribute to balance and gait in children with CP, this pilot study suggested that RAT is a feasible treatment for children with CP to improve balance and gait. The RAT could be deployed as an in-home telehealth treatment with its portability and user-friendly function [14]. However, the study was limited in several aspects. First, this study included a rather small convenience sample size within a diverse population from a single site. Second, we only treated a single limb, even in diplegic patient cases. Third, the current study focused on clinical and biomechanical measurements, not on muscle morphology, while other studies utilized ultrasound images and electromyography (EMG) to detect changes in lengthening profiles of muscle fascicles under passive or active ankle stretching interventions as the outcomes [36,37], or by applying advanced gait analysis [38]. Recently, Kalkman et al. discussed the effects of traditional stretching through physical therapy on the interactions between muscle and tendon in children with CP, and a potential strategy to improve the treatment [39].

Sensory and motor impairments in children with CP are related and complicated. Therefore, further studies with a large sample size, control group and follow-up evaluation post training would help to better understand the contributions of the different factors in the rehabilitation of CP. Furthermore, prospective studies need to explore the duration of improvement maintenance and potential value of continued or intermittent retraining on maintaining or further enhancing function. This could be carried out by assessment with combined technologies such as ultrasound images and 3D motion analysis with virtual reality on sensory and motor performance.

## 5. Conclusions

The robotic ankle training showed promise for being an effective therapeutic intervention for children with CP, by improving sensorimotor functions of the lower extremities. The robotic training provided benefit with high repetitions, visual integration with real-time feedback in a motivating therapy to engage children with CP in rehabilitation, leading to improvements in sensorimotor performance.

## Figures and Tables

**Table 1:** Demographics and characteristics of the study participants who completed the training program.

ID	Gender	Age (Years)	Height (m)	Weight (kg)	Training Side	GMFCS	Topographical Classification
1	M	8	1.38	33.3	Right	I	Hemiplegia
2	F	6	1.16	16.3	Right	II	Diplegia
3	F	11	1.35	30.4	Right	I	Hemiplegia
4	F	12	1.47	36.9	Right	I	Diplegia
5	M	5	1.14	21.0	Right	II	Diplegia
6	M	6	1.17	21.5	Right	I	Diplegia
7	F	10	1.45	32.5	Right	III	Diplegia
8	M	7	1.25	26.7	Right	II	Diplegia
	4 boys 4 girls	8.1 ± 2.6	1.29 ± 0.13	27.3 ± 7.2	Right	I (*n* = 4) II (*n* = 3) III (*n* = 1)	Hemi (*n* = 2) Di (*n* = 6)

Values are mean ± standard deviation.

**Table 2:** Proprioceptive acuity among the children with CP and the typically developing children.

Proprioception Test	Children with CP		TDC
	Pre	Post	
Proprioceptive Acuity (deg)			
Dorsiflexion	3.60 ± 2.38	3.08 ± 2.07	0.94 ± 0.43 *
Plantar flexion	−3.72 ± 2.38	−2.59 ± 1.94	−0.86 ± 0.48 *
Number of wrong answers			
Dorsiflexion	0.38 ± 1.06	0.25 ± 0.71	0.00 ± 0.00
Plantar flexion	1.00 ± 1.31	0.25 ± 0.71	0.00 ± 0.00

Values are mean ± SD. The star (*p* < 0.05) shows significant difference in proprioceptive acuity level between the children with CP (Pre-training) vs. typically developing children, TDC (Wilcoxon rank sum test). (*) indicates significant difference (*p* < 0.05).

**Table 3:** Clinical evaluation among the children with CP between pre and post training.

Outcome Measures	Pre	Post
PBS (0–56)	48.14 ± 6.67	49.57 ± 6.29 *
MAS (0–4)	1.38 ± 0.69	1.21 ± 1.04
SCALE (0–10)	7.14 ± 1.95	8.00 ± 2.00
TUG (sec)	11.08 ± 4.91	9.30 ± 4.14 *
6 MWT (m)	371.76 ± 4.91	402.72 ± 5.10 *
10 MWT (sec)	10.56 ± 1.59	9.21 ± 1.48

Values are mean ± standard deviation. PBS, 0–56 points; MAS, 0–4 points; SCALE, 0–10 points. * *p* < 0.05 for pre vs. post. Wilcoxon signed rank test.

## Data Availability

The data presented in this study are available on request from the corresponding author.
